# The Impact of Lethal Means Counseling on Openness to Out‐of‐Home Firearm Storage: Results From Project Safe Guard

**DOI:** 10.1111/sltb.70135

**Published:** 2026-08-13

**Authors:** Michael D. Anestis, Jennifer Paruk, Allison E. Bond, Nicholas Allan, Craig J. Bryan, Daniel W. Capron, AnnaBelle O. Bryan, Daniel Semenza

**Affiliations:** ^1^ New Jersey Gun Violence Research Center Piscataway New Jersey USA; ^2^ Rutgers School of Public Health Piscataway New Jersey USA; ^3^ University of Michigan Ann Arbor Michigan USA; ^4^ Ohio State University Wexner Medical Center Columbus Ohio USA; ^5^ University of Vermont Burlington Vermont USA; ^6^ Louisiana State University Baton Rouge Louisiana USA

## Abstract

**Introduction:**

Firearm access increases suicide risk and lethal means safety (LMS) counseling is widely promoted to reduce this risk by encouraging voluntary limitations on firearm access during crises. Although LMS has been shown to increase secure in‐home firearm storage, less is known regarding its ability to promote temporary out‐of‐home storage—an approach that may more clearly prevent suicide. This study evaluated whether Project Safe Guard (PSG), a brief LMS intervention, increases willingness to store firearms outside the home during periods of heightened distress.

**Method:**

Participants were 232 Mississippi National Guard members with home firearm access enrolled in the original PSG randomized trial. Individuals received either a single session of PSG or a time‐matched control condition. Willingness to store firearms outside the home during personal or household distress was assessed pre‐intervention, post‐intervention, and at 3 and 6‐month follow‐ups. Repeated‐measures ANOVAs tested change over time and the impact of condition.

**Results:**

Participants receiving PSG showed significantly greater increases in willingness to store firearms outside the home when distressed, with effects more robust immediately post‐treatment and in analyses focused on participants' own distress.

**Conclusion:**

PSG increases openness to temporary out‐of‐home storage and may represent a promising firearm suicide prevention strategy.

**Trial Registration:**

ClinicalTrials.gov ID: NCT03375099

## 1 Introduction

Because suicide risk can fluctuate rapidly, the availability of highly lethal means such as firearms increases the risk of suicide (Barnard et al. 2021). Approximately 90% of suicide attempts with a firearm are fatal (Conner et al. 2019), and individuals living in households with firearms have significantly higher odds of suicide compared to those living in households without firearms (Anglemyer et al. 2014).

Lethal Means Safety (LMS) counseling is a widely endorsed intervention to prevent suicide (U.S. Department of Veterans Affairs 2018) and addresses the reality that approximately one‐third of U.S. adults personally own a firearm (Pew Research Center 2024). LMS conversations encourage individuals to voluntarily reduce their access to firearms and other highly lethal means, especially during times of acute risk. LMS conversations are ideally held proactively before periods of distress, enabling firearm owners to implement these safety measures when needed.

LMS conversations encourage firearm owners to decrease their access to firearms through a variety of ways, including through secure firearm storage practices at home. Storing firearms locked, unloaded, and separate from ammunition provides firearm owners a chance to pause before accessing their firearms and ammunition during a suicidal crisis. Secure firearm storage practices also allow a spouse or other adult household member to temporarily restrict access to firearms during a crisis, such as when a firearm owner requests that their spouse change the combination to a safe until the crisis has passed. Project Safe Guard (PSG), a LMS intervention, has been shown to increase secure firearm storage practices with military service members (Anestis et al. 2021) and has since been adapted for broad implementation within the National Guard (Walsh et al. 2024).

However, it is essential to determine if LMS conversations influence firearm‐related outcomes beyond secure firearm storage at home. The current research on the relationship between secure firearm storage practices and adult suicide is limited. Several research studies, all using the 1993 National Mortality Follow back Survey, found that among adults who died by suicide, secure storage measures were not associated with whether a firearm was used in the suicide (Dahlberg et al. 2004; Miller et al. 2025; Shenassa et al. 2004). However, Shenassa et al. (2004) found in their analyses of decedents that secure firearm storage was in fact associated with decreased odds of dying by firearm suicide specifically among individuals who did *not* endorse an intention to die within the month before their death. Similarly, secure firearm storage was associated with decreased odds of dying by firearm suicide among those who had not used alcohol, not had an employment change, and not visited a mental health professional within the past year. These findings imply that secure storage did indeed reduce risk of firearm suicide among those without prior intention or within the context of other precipitating factors, suggesting it may have interrupted other more impulsive pathways towards firearm suicide. Another study found that U.S. Army soldiers who died by suicide were more likely to store a firearm at home loaded than propensity‐matched controls, though the use of firearm safety locks did not differ between the two groups (Dempsey et al. 2019). A third study that gathered data on suicide decedents via a sample of suicide loss survivors found that suicide decedents who stored their firearms securely in the home were less likely than those who stored their firearms unsecured to have utilized a firearm in their suicide death (Anestis et al. 2017). The mixed findings have prompted some researchers to question the utility of secure firearm storage as a firearm suicide prevention intervention (Miller et al. 2025); however, no studies have been equipped with the appropriate data to determine how many suicides were prevented entirely by the use of secure firearm storage, in or outside of the home, leaving the impact of such behaviors unknown. Although prior work has considered related questions, none have assessed the degree to which individuals opt against attempting suicide entirely or switch to a less deadly method and ultimately survive because their firearms were stored securely and thus less readily available in a moment of crisis. Since more research is needed to understand whether and how within‐home secure firearm storage prevents firearm owners from attempting suicide, it is imperative to examine how LMS influences additional methods of reducing firearm access, including storage outside the home. In answering this latter question, the utility of current LMS interventions in facilitating a less ambiguously effective form of firearm suicide prevention can be more clearly assessed. It should be noted that, although numerous efforts have been launched to promote out‐of‐home secure firearm storage, there is also a lack of data clearly demonstrating that voluntarily removing a firearm from the home during times of stress prevents death by suicide. That said, research on compulsory removal of firearms via extreme risk protection orders has demonstrated that removing firearms from the home in a moment of acute risk can be effective in preventing suicide (Miller et al. 2024; Swanson et al. 2024, 2017; Kivisto and Phalen 2018). This provides an empirical rationale for promoting out‐of‐home storage during times of risk. If LMS conversations only prompt increased within‐home secure firearm storage and the suicide prevention value of within‐home secure firearm storage is open to question, the utility of such interventions thus depends in part on their capacity for prompting storage away from home during times of crisis.

LMS conversations, in addition to promoting secure storage, also promote voluntary and temporary out‐of‐home firearm storage during times of increased risk. Firearm owners can temporarily store their firearms with family and friends, firearm retailers, shooting ranges, law enforcement agencies, and at self‐storage facilities, although the availability of these storage options vary by state law (McCourt et al. 2017). Although evidence on the impact of secure firearm storage on adult suicide risk requires additional investigation, research suggests that the removing firearms from the home could reduce the risk of suicide, as individuals in households without firearms have lower odds of suicide (Anglemyer et al. 2014).

Recent policies and programmatic efforts have supported both firearm retailers and firearm owners in expanding voluntary, temporary out‐of‐home storage. Some states have decreased liability for retailers offering temporary storage (LA§ 40:1800 2022), and have required that retailers accept the storage of a firearm during periods of crisis or heightened risk for suicide (California SB368 2024). Multiple organizations, such as Pause to Protect and The Armory Project, also support retailers in offering temporary storage services (Constans et al. 2023; Pause to Protect 2024; Wright‐Kelly et al. 2024). Organizations have also created maps for firearm owners to use to find locations that offer temporary storage (Betz et al. 2023; Gun Storage Map, n.d.). However, a substantial portion of firearm owners report they are not willing to use temporary out‐of‐home firearm storage, indicating that LMS conversations could play a substantial role in increasing acceptability for this safety measure. In a national study of firearm owners and their families, only 41% were willing in at least one circumstance to consider storing with a firearm retailer and 34% with law enforcement (Paruk et al. 2025). A study of firearm owners and their families in Colorado and Washington found higher willingness for temporary storage with family (63%) and a self‐storage facility (52%), although this still leaves a substantial proportion of firearm owners who are unwilling to do this at all (Barnard et al. 2023). LMS conversations have the potential to increase the willingness of firearm owners to temporarily store their firearms outside of the home during periods of higher risk for suicide. However, it is unknown if LMS conversations are associated with a change in willingness for temporary out‐of‐home storage.

### 1.1 Current Study

LMS counseling is a widely promoted evidence‐based intervention to prevent suicide and encourages decreasing access to firearms and other lethal means. Given the limitations of current research on the relationship between secure firearm storage and suicide of adult firearm owners, it is important to examine whether LMS conversations are associated with willingness to use additional methods to limit firearm access in times of crisis. In this current study, we examine whether PSG is associated with a change in willingness to temporarily store firearms outside of the home.

## 2 Method

### 2.1 Participants and Procedures

Participants were 232 members of the Mississippi National Guard who endorsed home firearm access. Data were collected as part of the original Project Safe Guard (PSG) randomized controlled trial between 2018 and 2020 and a full description of the procedures can be found in Anestis et al. (2021). All procedures were approved by the appropriate university institutional review boards and the US Army Medical Research and Materiel Command Human Research Protection Office. Participants received $50 for completing the baseline appointment and $75 for each follow‐up appointment. For the purpose of these analyses, individuals were classified as having been randomized to either receive a single session of PSG (*n* = 114) or a single session of the control condition (*n* = 118). The active condition of PSG involved a brief (~15 min), culturally humble, guided discussion of firearm storage using a motivational interviewing framework. The control condition, like PSG, utilized a motivational interviewing framework, but focused instead on different aspects of health and home safety (e.g., stress management, sleep hygiene).

### 2.2 Primary Outcome Variables

Participants were asked about their willingness to voluntarily store firearms away from home during times of distress through two separate questions, each of which served as the dependent variable in separate analyses. Participants were asked these two questions immediately before and after treatment, and 3 and 6‐months post‐treatment. The first question asked, “Are you open to the idea of letting someone you trust temporarily keep your gun for you if you become highly distressed?” The second question asked, “Are you open to the idea of letting someone you trust temporarily keep your gun for you if somebody you live with becomes highly distressed?” Responses were scored on the following 5‐point Likert scale: 0 = Not at all open, 1 = Somewhat open, 2 = Moderately open, 3 = Very open, 4 = Extremely Open.

### 2.3 Data Analytic Plan

Both outcomes were analyzed using piecewise categorical latent growth curve models in Mplus. This approach was selected because the repeated outcomes were assessed on 5‐point ordered categorical scales, making methods that assume continuous, normally distributed outcomes less appropriate. In addition, the study design made it plausible that any intervention‐related effect would emerge immediately following treatment and then either persist or attenuate over follow‐up. The piecewise specification therefore allowed us to separate the initial post‐intervention shift from subsequent follow‐up change.

For each outcome, the four repeated assessments, baseline, post‐intervention, 3‐month follow‐up, and 6‐month follow‐up, were modeled as ordered categorical indicators using a probit link and weighted least squares estimation with mean and variance adjustment (WLSMV). Growth was represented by an intercept factor, a treatment slope factor indexing the shift from baseline to post‐intervention, and a maintenance slope factor indexing subsequent change across follow‐up. Treatment condition was entered as a predictor of both slope parameters to test whether PSG was associated with the immediate post‐treatment shift and with later change over time.

To maintain a parsimonious and stable specification, thresholds were constrained to equality across time and slope variances were fixed to zero. Accordingly, the final models estimated average treatment‐related change over time rather than between‐person heterogeneity in growth parameters. The final convergence‐focused specification used THETA parameterization and a constrained baseline formulation.

As sensitivity analyses, we also examined dichotomous rates of any willingness at each assessment wave and described follow‐up movement among participants who reported no openness at baseline. These supplemental analyses were intended to clarify whether PSG increased the probability of endorsing any willingness to temporarily store firearms outside the home and whether it shifted participants who were initially fully resistant to out‐of‐home storage.

## 3 Results

**FIGURE 1:**
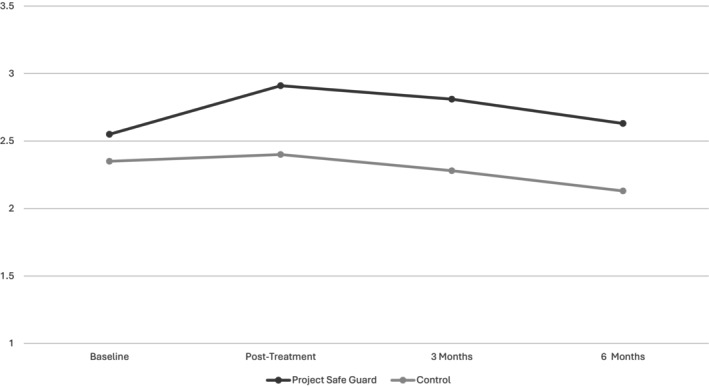
Scores at each time point for each treatment condition on willingness to store firearms temporarily away from home to prevent one's own suicide.

Descriptive statistics are presented in Table 1. Primary analyses used piecewise categorical latent growth models to examine whether PSG predicted an immediate post‐intervention shift in openness to temporary out‐of‐home firearm storage and whether it predicted subsequent change across follow‐up. For willingness to temporarily store firearms away from home if the participant became highly distressed, treatment condition significantly predicted the treatment slope, *b* = 0.67, SE = 0.23, *p* = 0.003, 95% CI [0.23, 1.11], indicating that participants randomized to PSG showed a greater immediate post‐intervention increase in openness than those in the control condition. In contrast, treatment condition did not significantly predict the maintenance slope, *b* = 0.13, SE = 0.31, *p* = 0.680, 95% CI [−0.47, 0.73], suggesting that the intervention did not alter the subsequent rate of change across follow‐up. The mean of the treatment slope was not significantly different from zero, *b* = −0.14, SE = 0.13, *p* = 0.300 (Table 2; Figure 1).

**TABLE 1:** Descriptive statistics for the sample.

	% (*n*)
Sex	
Male	87.5 (203)
Female	12.5 (29)
Race	
White	77.2 (179)
Black	22.0 (51)
American Indian/Alaskan Native	1.3 (3)
Asian	0.9 (2)
Other	0.4 (1)
Hispanic	3.1 (7)
Relationship status	
Married, living together	57.8 (134)
Single	25.9 (60)
Cohabitating	3.4 (8)
Married, geographically separated	2.6 (6)
Divorced/separated	10.3 (24)

**TABLE 2:** Primary and model effects for out‐of‐home storage willingness.

Outcome	Parameter	Estimate	SE	95% CI	*p*
Self‐distress	Intercept mean	3.629	0.475	[2.70, 4.56]	< 0.001
Self‐distress	Treatment slope mean	−0.137	0.133	[−0.40, 0.12]	0.300
Self‐distress	Group → treatment slope	0.669	0.226	[0.23, 1.11]	0.003
Self‐distress	Group → maintenance slope	0.127	0.307	[−0.47, 0.73]	0.680
Household‐distress	Intercept mean	3.975	0.539	[2.92, 5.03]	< 0.001
Household‐distress	Treatment slope mean	−0.160	0.142	[−0.44, 0.12]	0.259
Household‐distress	Group → treatment slope	0.468	0.234	[0.01, 0.93]	0.046
Household‐distress	Group → maintenance slope	0.136	0.320	[−0.49, 0.76]	0.670

*Note:* Estimates are unstandardized probit coefficients from the primary ordinal piecewise categorical growth models. The treatment slope indexes the immediate shift from baseline to post‐intervention. The maintenance slope indexes subsequent change across follow‐up. Thresholds were constrained equal across time, and slope variances were fixed to zero. Confidence intervals were calculated from the reported estimates and standard errors.

**FIGURE 2:**
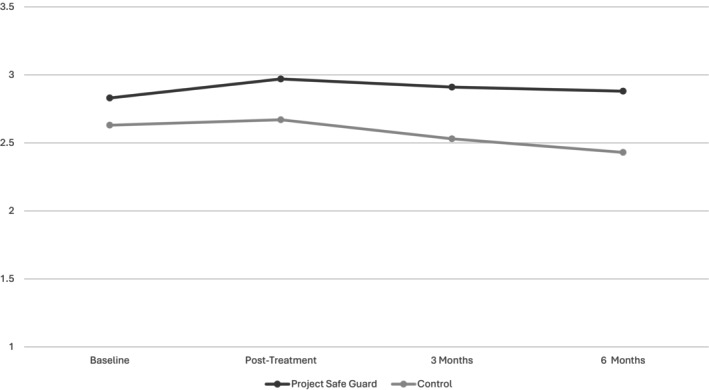
Scores at each time point for each treatment condition on willingness to store firearms temporarily away from home to prevent the suicide of someone else in the home.

A similar pattern emerged for willingness to temporarily store firearms away from home if somebody living with the participant became highly distressed. Treatment condition significantly predicted the treatment slope, *b* = 0.47, SE = 0.23, *p* = 0.046, 95% CI [0.01, 0.93], indicating a modest but statistically significant immediate post‐intervention increase in openness among participants assigned to PSG relative to control. Treatment condition again did not significantly predict the maintenance slope, *b* = 0.14, SE = 0.32, *p* = 0.670, 95% CI [−0.49, 0.76], indicating no evidence that the intervention changed the subsequent follow‐up trajectory. The mean of the treatment slope was also not significantly different from zero, *b* = −0.16, SE = 0.14, *p* = 0.259. Taken together, these findings suggest that PSG was associated with an early upward shift in openness for both outcomes, but not with additional divergence in change across follow‐up (Table 2; Figure 2).

The baseline‐zero mover analyses further clarified the pattern of results by examining only participants who endorsed no willingness at baseline. For the self‐distress outcome, among participants who endorsed no openness at baseline and had observed follow‐up data, a larger proportion of PSG participants than control participants reported any willingness immediately following the intervention, 56.50% versus 20.80%, risk difference = 35.70 percentage points, 95% CI [9.70, 61.70], Fisher's exact *p* = 0.017. At 3 months, the corresponding rates were 68.40% versus 36.40%, risk difference = 32.10 percentage points, 95% CI [3.10, 61.10], Fisher's exact *p* = 0.062. At 6 months, rates were 61.10% versus 40.90%, risk difference = 20.20 percentage points, 95% CI [−10.30, 50.70], Fisher's exact *p* = 0.341. Thus, for the self‐distress outcome, the clearest between‐group difference among initially resistant participants was observed immediately following treatment, with a similar but weaker pattern at 3 months (Table 3).

**TABLE 3:** Baseline‐zero mover analysis among participants with observed follow‐up.

Outcome	Follow‐up	Control moved/observed (%)	PSG moved/observed (%)	Risk difference % points	95% CI	Fisher's exact *p*
Self‐distress	Post	5/24 (20.8%)	13/23 (56.5%)	35.7	[9.7, 61.7]	0.017
Self‐distress	3‐month	8/22 (36.4%)	13/19 (68.4%)	32.1	[3.1, 61.1]	0.062
Self‐distress	6‐month	9/22 (40.9%)	11/18 (61.1%)	20.2	[−10.3, 50.7]	0.341
Household‐distress	Post	3/17 (17.6%)	7/14 (50.0%)	32.4	[0.5, 64.2]	0.121
Household‐distress	3‐month	5/16 (31.2%)	6/10 (60.0%)	28.7	[−9.2, 66.7]	0.228
Household‐distress	6‐month	7/16 (43.8%)	6/9 (66.7%)	22.9	[−16.3, 62.2]	0.411

*Note:* This analysis was restricted to participants who endorsed no willingness at baseline and had observed data at the relevant follow‐up. Risk differences are reported in percentage points.

For the household‐distress outcome, the same directional pattern was present but inferential support was weaker. Among baseline‐zero participants with observed follow‐up, 50.00% of PSG participants versus 17.60% of control participants reported any willingness at post‐intervention, risk difference = 32.40 percentage points, 95% CI [0.50, 64.20], Fisher's exact *p* = 0.121. At 3 months, the corresponding rates were 60.00% versus 31.20%, risk difference = 28.70 percentage points, 95% CI [−9.20, 66.70], Fisher's exact *p* = 0.228. At 6 months, rates were 66.70% versus 43.80%, risk difference = 22.90 percentage points, 95% CI [−16.30, 62.20], Fisher's exact *p* = 0.411. Taken together, these analyses suggest that PSG may have shifted a meaningful proportion of initially resistant participants upward, particularly immediately following treatment, although this pattern was statistically clearest for the self‐distress outcome.

## 4 Discussion

The present study builds on prior research demonstrating the effectiveness of Project Safe Guard (PSG) in promoting secure firearm storage in the home (Anestis et al. 2021) and examines its impact on willingness to store firearms outside the home during periods of heightened distress. Results indicated that PSG increased participants' willingness to store a firearm outside the home, particularly if they themselves became highly distressed. Both in the full sample and among those who initially expressed zero willingness to utilize out‐of‐home storage, PSG prompted immediate increases in openness, with findings more robust within the initially unwilling subsample and in analyses focused on the participant's own distress. These findings suggest that a brief lethal means safety counseling intervention can meaningfully increase willingness to store firearms outside the home, which may, in turn, help reduce suicide risk.

Relative to those in the control condition, participants who received PSG were significantly more willing immediately post‐treatment to store firearms away from home if they themselves became distressed. Treatment condition was not associated with changes in willingness at the follow‐up assessments, indicating that PSG did not have an increasingly more potent impact on willingness to store away from home at follow‐up relative to control, although the initial differences remained intact over time. A similar pattern emerged in the analysis examining willingness to store firearms away from home should another household member become distressed, although the magnitude of those findings was smaller.

Our baseline‐zero mover analyses—which considered the degree to which the treatment conditions resulted in increased openness among individuals initially completely unwilling to utilize out‐of‐home storage—provided promising preliminary evidence that PSG can be impactful among those resistant to the practice. Here again, the findings were most pronounced immediately post‐treatment and when focused on the individual's own distress, with 56.5% of those in PSG exhibiting some willingness, relative to 20.8% within the control group. The between group effect weakened at 3 months (68.4% vs. 36.4%) and dissipated by 6 months (61.1% vs. 40.9%), although not because of a return to unwillingness among those who received PSG. The effects were statistically non‐significant in the analyses examining out‐of‐home storage in response to household distress; however, the pattern of the findings was fairly consistent with those from the analyses considering participants' own distress (post‐treatment: 50.0% vs. 17.6%; 3‐months: 60.0% vs. 31.2%; 6‐months: 66.7% vs. 43.8%).

Taken together, these findings provide promising preliminary evidence that an intervention focused on secure firearm storage and designed to work within the value system and home situation of the firearm owner, even without systematically promoting out‐of‐home storage, may increase willingness to use out‐of‐home firearm storage. The effects were more pronounced when focused on participants' own distress, which contrasts with prior research demonstrating that firearm owners are generally more open to within home firearm storage to reduce household suicide risk (e.g., Anestis et al. 2019) and also became less pronounced as time passed. Given that the intervention involved a single brief conversation, administered in a primary prevention approach and with out‐of‐home storage not systematically embedded within the protocol, the diminishing effects over time are not entirely surprising. In all likelihood, in order to facilitate larger and more sustained effects, the dosage of the intervention would need to be increased through changes such as ensuring that out‐of‐home storage is discussed in all PSG sessions or incorporating booster sessions. Additionally, broader environmental changes may help decrease initial obstacles to openness and, in doing so, set the stage for a more pronounced response. For instance, messaging campaigns that normalize the concept of out‐of‐home storage and increase familiarity with the concept may cause some of the initial hesitance seen in this sample to dissipate. Removing logistical barriers, such as legislation in some states (e.g., New Jersey) that prevents temporarily storing firearms with friends and family may cause some individuals who do not want to store their firearms with retailers or law enforcement to become more open and respond more fully to PSG.

Overall, the results from this study provide a promising path forward for the field of firearm suicide prevention. Most firearm owners are unwilling to store a firearm outside of the home (Paruk et al. 2025), and yet out‐of‐home storage is considered one of the most effective strategies for preventing firearm suicide. Demonstrating that a brief lethal means safety intervention can increase willingness to employ out‐of‐home storage strategies during times of crisis is therefore encouraging. Findings from this study highlight the importance of disseminating lethal means trainings—such as PSG—broadly, given their ability to promote out‐of‐home storage and increase overall willingness to adopt secure storage practices. Scaling to other branches of the military and civilian populations can potentially yield much broader uptake of secure storage efforts to reduce population suicide risk. Additionally, while significant funding has been invested in initiatives such as firearm storage maps, Pause to Protect, and the Armory Project, research suggests that out‐of‐home storage remains relatively uncommon. Disseminating PSG and other lethal means counseling interventions that increase willingness to store firearms outside the home could enhance the reach and effectiveness of these ongoing resource‐based initiatives that provide options to those looking to store their firearms away from home.

Although novel, the present study is not without limitations. First, the study was conducted among Mississippi National Guard members—a unique subset of the population—and findings may not be generalizable to active‐duty service members or civilians. Indeed, the lack of legal barriers to temporarily transferring firearms to friends and family within Mississippi may have explained the relatively high levels of initial willingness in this sample and could differ from what is seen in more restrictive states that would require out‐of‐home storage to take place in specific venues (e.g., firearm retailers, law enforcement agencies). Although it is unclear to what extent residents of Mississippi or other states are aware of local restrictions related to temporary firearm transfers, it is reasonable to assume that this could function as a barrier to individuals where regulations are more restrictive. This highlights the importance of the work of groups like the Armory Project, who are creating infrastructures across several states to facilitate viable out‐of‐home storage options for firearm owners. Ongoing PSG trials being conducted outside of Mississippi should afford an opportunity to test the impact of this limitation. Second, while the study measured willingness to store firearms outside the home, it did not assess if individuals actually engaged in storing their firearms outside of the home. Given that the trial was a primary prevention trial, risk acuity was low and the base rate of suicidal crises precluded an assessment of such behaviors. Third, we do not know what situations (e.g., personal experience) increased willingness to store outside of the home. Future research should explore actual engagement in our of the home storage, and examine factors and experiences that influence individuals' willingness to store firearms outside of the home.

This study provides evidence that Project Safe Guard—a brief lethal means counseling intervention—increases willingness to store firearms outside the home during times of personal or family distress, and that these effects were sustained over time. These findings offer a path forward for the field of firearm suicide prevention, suggesting that interventions promoting secure firearm storage can increase openness to storing firearms outside the home during periods of crisis; and such efforts may support broader prevention initiatives (e.g., safe storage maps).

## Author Contributions

**Michael D. Anestis:** conceptualization, investigation, funding acquisition, writing – original draft, methodology, writing – review and editing, formal analysis, project administration, supervision. **Jennifer Paruk:** writing – original draft, writing – review and editing. **Daniel W. Capron:** investigation, funding acquisition, writing – review and editing, methodology, supervision. **AnnaBelle O. Bryan:** funding acquisition, investigation, writing – review and editing, supervision. **Nicholas Allan:** formal analysis, writing – original draft. **Craig J. Bryan:** investigation, funding acquisition, writing – review and editing, methodology, supervision. **Daniel Semenza:** writing – original draft, writing – review and editing. **Allison E. Bond:** writing – original draft, writing – review and editing.

## Funding

This work was in part supported by the Military Suicide Research Consortium (MSRC), an effort supported by the Office of the Assistant Secretary of Defense for Health Affairs under Award No. (W81XWH‐16‐2‐0003). Opinions, interpretations, conclusions and recommendations are those of the authors and are not necessarily endorsed by the MSRC or the Department of Defense.

## Ethics Statement

All procedures were approved by the appropriate university institutional review boards and the US Army Medical Research and Materiel Command Human Research Protection Office.

## Consent

All participants provided written informed consent prior to participating in the protocol.

## Conflicts of Interest

Drs. Anestis and Bryan report personal income related to secure firearm storage in the form of book royalties, speaking fees, consulting fees, and training fees.

## Data Availability

Data can be made available upon request to the authors.
